# Resazurin assay for assessment of antimicrobial properties of electrospun nanofiber filtration membranes

**DOI:** 10.1186/s13568-019-0909-z

**Published:** 2019-11-13

**Authors:** Eva Travnickova, Premysl Mikula, Jakub Oprsal, Marie Bohacova, Lubomir Kubac, Dusan Kimmer, Jana Soukupova, Michal Bittner

**Affiliations:** 1 RECETOX Centre, Faculty of Science, Masaryk University, Kamenice 5, 625 00 Brno, Czechia; 2 Department of Veterinary Public Health and Forensic Medicine, University of Veterinary and Pharmaceutical Sciences Brno, Palackeho 1946/1, 612 42 Brno, Czechia; 3 SYNPO a. s., Pardubice, S. K. Neumanna 1316, 532 07 Pardubice, Czechia; 4 Centre for Organic Chemistry Ltd., Rybitvi 296, 533 54 Rybitvi, Czechia; 5 Centre of Polymer Systems, University Institute, Tomas Bata University, Trida Tomase Bati 5678, 760 01 Zlin, Czechia; 6 Regional Center of Advanced Technologies and Materials, Department of Physical Chemistry, Palacky University, Slechtitelu 27, 783 71 Olomouc, Czechia

**Keywords:** Resazurin, Antimicrobial activity, Electrospun nanofiber filtration membranes, Silver nanoparticles, Quaternary ammonium salts

## Abstract

We developed a simple and fast microplate assay for evaluation of the antimicrobial activity of electrospun nanofiber filtration membranes or similar porous materials for water treatment technologies. Resazurin (alamarBlue^®^) was used as an indicator of the amount of viable experimental microorganisms Gram-negative *Escherichia coli*, Gram-positive *Enterococcus faecalis,* and natural wastewater treatment plant effluent bacteria. A bacterial inoculum of concentration 1–3 × 10^5^ CFU mL^−1^ was pipetted onto the surface of assessed both functionalized and respective control membranes and incubated in 12-well plates for 4 h at 37 °C. Kinetics of resazurin metabolization, i.e. its reduction to fluorescent resorufin, was evaluated fluorimetrically (λ_ex_520/λ_em_590 nm). A number of viable bacteria on the membranes expressed as CFU mL^−1^ was calculated from the kinetic curves by using calibration curves that were constructed for both experimental bacterial species. Antimicrobial activities of the membranes were evaluated by either resazurin assay or modified ISO 20743 plate count assay. Results of both assays showed the significant antimicrobial activity of membranes functionalized with silver nanoparticles for both bacterial species and wastewater treatment plant effluent bacteria as well (log CFU reduction compared to control membrane > 4), while membranes containing specific quaternary ammonium salts were inefficient (log CFU reduction < 1). The suitability of resazurin microplate assay for testing nanofiber filtration membranes and analogous matrices has proven to be a faster and less demanding alternative to the traditionally used approach providing comparable results.

## Introduction

Membrane filtration has recently become a good alternative to more commonly used water purification technologies based on chlorination or oxidation processes, mainly due to their reducing costs and high microbial removal efficacy. Besides other commercially available filtration membranes, electrospun nanofiber membranes have great potential, since they represent even more promising choices for high flux microfiltration applications (Kaur et al. 2014; Lev et al. 2014; Seyed Shahabadi et al. 2016; Wang et al. 2012). Moreover, either polymer matrix for nanofiber preparation or surface of the pristine membrane itself can be functionalized with various techniques or antimicrobial agents that suppress biofilm formation, i.e. biofouling (reviewed in Zhu et al. 2018).

Recently, the biofouling control has been a challenge, as growing biofilms may affect filtration performance with the need for membrane cleaning or replacement (Iorhemen et al. 2017). The following agents, for direct inhibition of microorganism growth, have been successfully used for functionalization of nanofiber membranes: titanium dioxide (Daels et al. 2015; He et al. 2017; Si et al. 2017), silver nanoparticles (AgNPs) (He et al. 2017; Liu et al. 2018; Zhao et al. 2017), quaternary ammonium salts (QAS) (Park and Kim 2017) and some others (Coelho et al. 2017; Si et al. 2017; Zhu et al. 2017). Indirect antifouling surface modification methods blocking initial microbial adhesion to the membrane have also been described (Bazaka et al. 2012; Yang et al. 2014) and even an approach combining both direct and indirect antimicrobial/antifouling actions has recently been introduced (Goetz et al. 2016).

Antimicrobial/antifouling effects of filtration membranes can be evaluated by various analytical techniques. As an example, standard plate counts i.e. a number of colonies of viable cultivable microorganisms grown on agar media have been commonly used for this purpose as it is undemanding for special equipment and well-established. This method enables to focus on specific groups of bacteria (e.g. potential pathogens), moreover, well-standardized protocols are available (Allen et al. 2004; Ristic et al. 2011; Van Nevel et al. 2017). Nevertheless, plate counting has several drawbacks: it is time-consuming, demanding, and the results are usually delivered later, due to long incubation time. This is especially true with slowly growing natural microbial communities cultivated on minimal media that may require a long incubation period of 7 days (Van Nevel et al. 2017). Moreover, only a small fraction of environmental bacteria can be grown on agar plates due to the viable but not cultivable physiological state they may possess or even their uncultivability under laboratory conditions (Staley and Konopka 1985; Van Nevel et al. 2017). Other potentially suitable methods involve atomic force microscopy (James et al. 2017), advanced imaging techniques such as confocal laser scanning microscopy (Neu et al. 2010; Nguyen et al. 2012) or electron microscopy (Nguyen et al. 2012; Wagner and Horn 2017), yet they are still quite expensive, demanding and require advanced instrumentation and experienced handling.

Here we present results of another approach based on quantification of bacteria using in situ metabolizations of resazurin. Resazurin or alamarBlue^®^ is a well-known indicator dye for the assessment of viability in both microbial and cell culture applications. Resazurin has already been used e.g. for the detection of microbial contamination of milk or determination of chemical cytotoxicity and minimum inhibitory concentration values of certain antibiotics (Bigalke 1984; Drummond and Waigh 2000; McNicholl et al. 2007; Sarker et al. 2007).

This paper represents probably the first application of resazurin assay for assessment of antimicrobial properties of electrospun nanofiber membranes used in water treatment technologies. This assay was evaluated by using two bacterial species, Gram-negative *Escherichia coli* and Gram-positive *Enterococcus faecalis,* that are both common in aquatic ecosystems and water treatment technologies (Monticelli et al. 2019; Perkins et al. 2016; Pinto et al. 1999) as well as natural microbial communities from wastewater treatment plant (WWTP) effluent. The resazurin assay was performed in 12-well microplate setup and the recalculation of resulting fluorescent units to CFU mL^−1^ was done through the calibration curves that were established separately for both bacterial species. Finally, the resulting antimicrobial effects determined by the resazurin assay were compared with the results obtained by a modified ISO 20743 plate count assay.

## Materials and methods

### Preparation of electrospun nanofiber filtration membranes

**Fig. 1:**
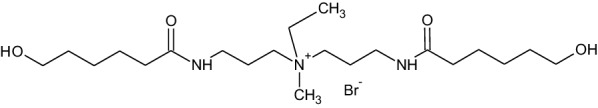
Chemical structure of QAS used for membrane functionalization

For fiber-forming polymer blend, isophorone diisocyanate with 1,6-hexanediol was used. During the synthesis of the polymer chain, formerly prepared QAS (systematic name bis{3,3′-[(6-hydroxyhexanoyl)amino]}-*N*-ethyl-*N*-methyldipropan-1-ammonium bromide, Fig. 1) was continuously added to the reaction mixture to concentration 5% (v/v). The product was soluted with dimethylformamide to 30% and processed via electrospinning equipment (Centre of Polymer Systems, Tomas Bata University, Zlin, Czechia) using multi-nozzle nanofiber forming jets. As support layers, polyurethane microfiltration reinforced foam (MFRF), polypropylene (PP) or viscose (VS) were used. Area weight of final nanofiber membranes together with support layers was 2.69, 2.56 and 2.69 g cm^−2^ respectively.

Membranes with AgNPs were prepared from polyurethane (PU) solution in dimethylformamide (DMF). PU/DMF mixture was pre-treated with a hyper branched polymer linker containing numerous N-terminated functional groups. The polymeric solution was processed using the same equipment and jets as QAS membranes. AgNPs were generated in one step and immobilized on the top of functional groups available from the polymer linker anchored into the fibers (Palacky University in Olomouc—Regional Centre of Advanced Technologies and Materials, Olomouc, Czechia). The particles were covalently bonded thanks to the interaction between the nitrogen from the linker and silver. The diameter of the generated particles was approximately 20 nm. Final membrane area weight was 2.44 g cm^−2^.

### Preparation of bacterial suspensions

Gram-negative bacteria *Escherichia coli* CCM 3954 and Gram-positive bacteria *Enterococcus faecalis* CCM 4224 were purchased from the Czech Collection of Microorganisms (Masaryk University, Brno, Czechia). These bacteria has been chosen as model microorganisms as they represent environmentally relevant species. The microorganisms were stored at − 80 °C in tryptic soy broth (TSB, Sigma-Aldrich Inc., USA) with 20% v/v of glycerol. Frozen stock cultures were used to prepare the first subculture by streaking onto tryptic soy agar (TSA, Sigma-Aldrich Inc., USA), which was then incubated for 24 h at 37 °C. After that, a second subculture was prepared by inoculating TSB with several colonies from the first subculture and incubated for 18 to 24 h at 37 °C on an orbital shaker at 110 rpm. Subsequently, 400 μL of the second subculture were transferred into 20 mL of TSB and incubated in the same way as the second subculture for 100 min to get bacteria in an exponential growth phase (Additional file 1: Material S1). The resulting microbial suspensions were used in subsequent assays.

WWTP effluent was taken on 8th of January 2019 just from the effluent pipe at the meeting point with Ochozsky potok. Location and details of the sample are described in Additional file 1: Material S2. The WWTP effluent was tested without filtration. The raw sample was mixed with TSB in the ratio of 1:1, and immediately used in subsequent assays.

### Resazurin reduction assay

#### Setup of calibration curves

**Fig. 2:**
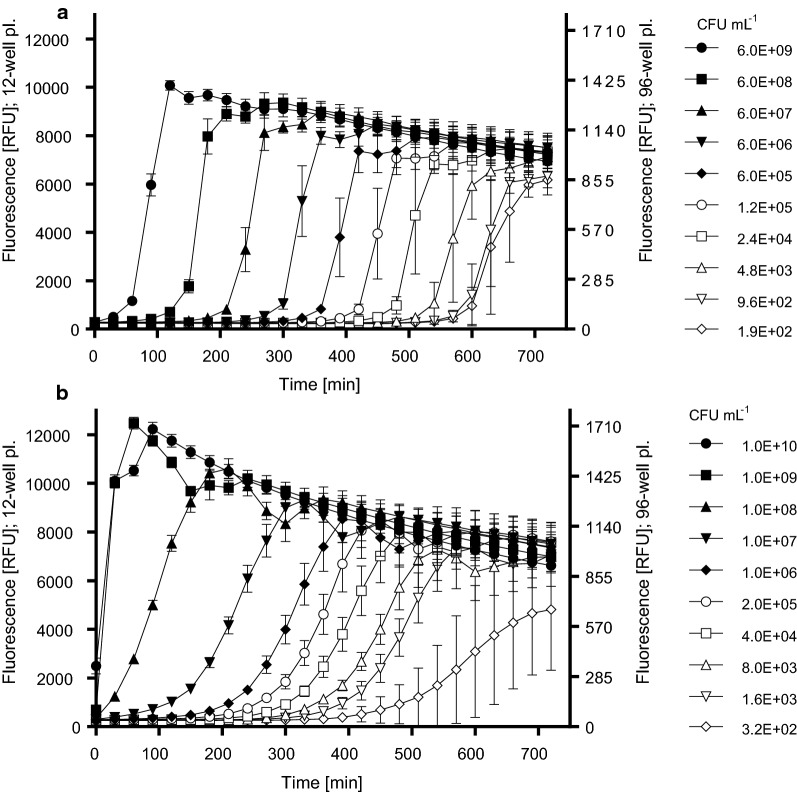
Kinetics of resazurin metabolization in bacterial suspensions of *E. coli* (**a**) and *E. faecalis* (**b**) having various bacterial concentrations. Values represent the mean ± SD of hexaplicate determinations. The figure shows a set of results of one experimental run as an example of three analogous independent experiments for both bacterial species

In order to define the relationship between the bacterial concentration in the sample suspensions and intensity of the resorufin fluorescence, resazurin metabolization curves were plotted for both bacterial species. The calibration curve describes a relationship between the concentration of bacteria (CFU mL^−1^) and the respective microbial-generated resorufin (RFU). The bacterial suspensions prepared according to the previous chapter were serially diluted five or tenfold dilution down to the lowest concentration of approximately 10^2^ CFU mL^−1^. The exact concentrations of suspensions (CFU mL^−1^) were calculated from the OD_600_ values, where OD_600_ = 1 corresponds to 2.3 × 10^9^ and 5.9 × 10^9^ CFU mL^−1^ for *E. faecalis* and *E. coli,* respectively. These values were determined in advance using the conventional plate counting on TSA. The resazurin metabolization experiments were performed in 96-well plates. A volume of 10 µL of each suspension concentration was mixed with 200 µL of resazurin at a concentration of 20 µmol L^−1^ in phosphate buffered saline (PBS). The fluorescence (RFU) of microbial-generated resorufin was recorded at λ_ex_ = 520 nm/λ_em_ = 590 nm immediately after the resazurin dosing to all wells, and then again in 30 min periods for 720 min using a multi-detection microplate reader Synergy 4 (BioTek Instruments Inc., USA). Each concentration level was measured in hexaplicate and the mean ± SD was calculated (Fig. 2). Three independent experiments were performed for both bacterial species. The T285 values (which equals to T2000 values in 12-well plates, see Additional file 1: Material S3), i.e. period to reach the RFU of 285, were calculated from the curves. The final calibration curve was constructed by plotting the T285 values (i.e. T2000 values in 12-well plates) against the corresponding CFU mL^−1^ values.

#### Evaluation of the antimicrobial activity of membranes by resazurin assay

Due to practical reasons, involving especially easier manipulation with membrane cuts, 12-well microplates, instead of 96-well ones, were used for the evaluation of antimicrobial activities of the membranes. Because of that, preliminary comparative experiments were conducted to characterize differences in resazurin metabolization in both types of microplates (Additional file 1: Material S3). As a result, RFU values of bacterial inoculum detected in 12-well plates were found seven times higher than in 96-well microplates. Therefore, T2000 fluorescence values were monitored in these experiments instead of T285 that were taken previously.

Square-shaped membrane cuts with the side-length of 1.3 cm were placed into 12-well plates being oriented with the nanofiber layer up and support layer down. Subsequently, they were inoculated with a 100 µL of bacterial suspension (either *E. coli* or *E. faecalis* in TSB) with the concentration adjusted to 1 to 3 × 10^5^ CFU mL^−1^, or raw WWTP effluent diluted 1:1 with TSB. Microplates were incubated for 4 h at 37 °C. Three types of controls were assessed simultaneously: (1) membrane cuts dosed with 100 µL of sterile TSB to check either possible bacterial contamination of membranes or the unfavorable reduction of resazurin by membranes themselves, (2) 100 μL of bacterial inoculum per well without membrane for confirmation of appropriate bacterial proliferation in inoculum, (3) 100 μL of sterile TSB without bacteria and membrane for evaluation of background fluorescence of consequently added resazurin.

After the incubation, 2 mL of resazurin at the concentration of 20 μmol L^−1^ in PBS tempered to 37 °C was added to all wells. Plates were incubated at 37 °C on an orbital shaker at 150 rpm for 30 min for *E. coli*, or 5 min for *E. faecalis* due to the faster resazurin metabolization by *E. faecalis*. Then, the membrane cuts were removed, and the fluorescence was measured at λ_ex_ = 520 nm/λ_em_ = 590 nm in 30 min periods for 720 min. The time to reach a signal of 2000 RFU was determined for each sample. At this time point, the linear part of the fluorescence curves started. Finally, the number of viable bacteria in each well was calculated using the equations of respective calibration curves. A number of bacteria in WWTP effluent sample was expressed in *E. coli* equivalents (EEQ), as a setup of a calibration curve of resazurin metabolization in WWTP effluent samples does not seem practical due to highly variable characteristics of different effluent samples. Thus, the *E. coli* calibration curve was used providing results in log CFU_EEQ_ mL^−1^. All samples and controls were tested in triplicates in three independent runs for each bacterial species.

### Plate count assay

The antibacterial activity of membranes was also evaluated according to ISO 20743 guideline “Textiles – Determination of the antibacterial activity of textile products” using the adapted absorption method. Square-shaped membrane cuts with the side-length of 1.3 cm were placed into sterile glass vials in two batches of triplicates for both functionalized and control membranes, inoculated by 100 μL of *E. coli* or *E. faecalis* suspension in TSB at a concentration of 1–3 × 10^5^ CFU mL^−1^. 10 mL of a transferring solution, prepared by dissolving of 8.5 g of sodium chloride and 2.0 g of Tween80 in 1 L of distilled water, was added immediately to the first batch of vials. They were sealed with sterile caps and vortexed 5 times for 5 s. The samples were subsequently serially diluted, plated on TSA and incubated for 18 to 24 h at 37 °C. The second batch of vials was sealed by sterile caps to prevent drying and incubated at 37 °C for 4 h. After incubation, the second batch was processed in the same way as the first one.

### Data analysis

For calibration curve fitting, RFU values of individual wells and corresponding time points were compiled together for each bacterial suspension. First, the curves of fluorescence versus time of incubation were analyzed by regressions using GraphPad Prism 7 (GraphPad Software, Inc., CA, USA), and a time to reach RFU 2000 (T2000) was calculated for each bacterial concentration level. Second, T2000 values were plotted against log CFU mL^−1^, and the calibration curve including confidence intervals with a confidence level of 95% was constructed using GraphPad Prism 7.

Data obtained in experiments evaluating the antimicrobial activity of the membranes by resazurin assay were reported as means of log CFU per mL ± SD of triplicate determination unless stated otherwise. Three independent experiments were carried out for all types of membranes, both bacterial species, and one WWTP effluent sample. The numbers of bacteria calculated from relevant calibration curves and expressed in CFU mL^−1^ were set up to formula Eq. 1, where log*C*_4h_ was a logarithm of average number of bacteria detected for control non-functionalized membrane samples after 4 h of incubation, while log*S*_4h_ was a logarithm of average number of bacteria found in samples of functionalized membranes after the same incubation period.

1 $$A = \log C_{{4{\text{h}}}} - \log S_{{4{\text{h}}}}$$

Antimicrobial activity (*A*) of functionalized membranes was expressed as a log reduction of bacterial number (in CFU mL^−1^) compared to control, non-functionalized membranes. The criterion of significance from the plate counting ISO 20743 method was adopted, where A < 2 means non-significant antimicrobial activity.

For plate count assay, colonies grown on agar plates were counted and the antibacterial activity of membranes was calculated as follows:

2 $$A = \left( {\log C_{{4{\text{h}}}} - \log C_{{0{\text{h}}}} } \right) - \left( {\log S_{{4{\text{h}}}} - \log S_{{0{\text{h}}}} } \right)$$

where log*C*_0h_ was a logarithm of an average number of colonies counted in control samples just after the inoculation, and log*S*_0h_ represented a logarithm of the average number of colonies, counted on functionalized membrane just after the inoculation. When the *A* < 2, the antimicrobial activity was considered insignificant. Numbers of bacterial colonies grown on agar plates corresponded to the volume of 100 µL of originally dosed inocula, thus the final concentration per mL was 10 times higher. The validation criteria stated in ISO standard for this assay were met, thus the experiment was performed only once for each bacterial species.

## Results

### Calibration curves—resazurin assay

The kinetics of resazurin transformation to resorufin in bacterial suspensions of both bacterial species are summarized in Fig. 2, where the resazurin metabolization curves for various initial concentrations of bacteria are depicted. The highest bacterial concentration almost immediately metabolized the resazurin with a steep increase in fluorescence, lower bacterial concentrations started to increase the amount of resorufin with a slower slope after a lag phase of variable duration.

**Fig. 3:**
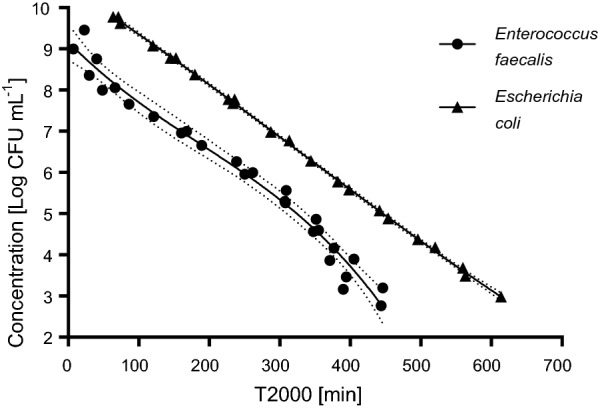
Calibration curves describing the relationship between the concentration of the bacteria in the wells and the rate of resazurin metabolization. Depicted results are T2000 values compiled from the three independent experiments for both *E. coli* and *E. faecalis*. Dotted lines depict confidence intervals with a confidence level of 95%. T2000 values under the LOQ level (i.e. 10^3^ CFU mL^−1^) are not depicted in the graph

The time points T285 (equal to T2000 in 12-well plates) were used for plotting calibration curves (Fig. 3). The calibration curve for *E. coli* is described by the equation y = – 0.125x + 10.604 that is linear over the range from 5.9 × 10^9^ to 9.4 × 10^2^ CFU mL^−1^ (concentration in the suspension) with the goodness of fit value R^2^ = 0.9993. The calibration curve for *E. faecalis* is characterized by the cubic equation y = − 6 × 10^−8^x^3^ + 3 × 10^−5^x^2^ − 0.0174x + 8.1754 over the range from 1.0 × 10^10^ to 1.6 × 10^3^ CFU mL^−1^ with the goodness of fit value R^2^ = 0.9778 (Fig. 3). LOQ of resazurin assay was set as the lowest point of calibration curves which is 10^3^ CFU mL^−1^ for both *E. coli* and *E. faecalis* bacteria species, which is equal to 10 CFU per sample because 0.01 mL of suspension per well was used in 96-well plates.

### Antimicrobial activity of membranes—resazurin assay

**Fig. 4:**
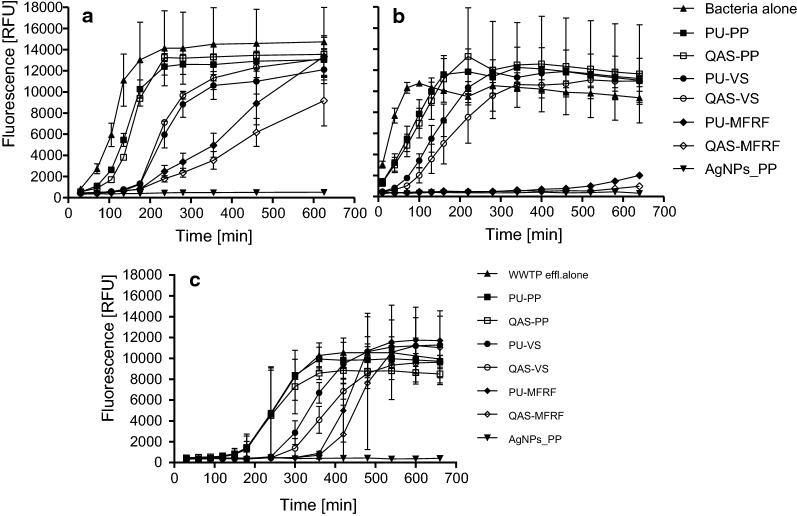
Resazurin metabolization curves for *E. coli* (**a**), *E. faecalis* (**b**), and WWTP effluent (**c**) incubated with various membranes on PP, VS and MFRF supporting layers. Terms “Bacteria alone” and “WWTP effl. alone” mean pure inoculum incubated without a membrane. Values represent the mean ± SD of triplicate determinations. This figure shows a set of results of one experimental run as an example of three analogous independent experiments for either *E. coli*, *E. faecalis* or WWTP effluent

The resazurin metabolization curves of bacteria incubated with QAS-functionalized, AgNPs-functionalized, and respective control membranes are depicted for both *E. coli* and *E. faecalis* as well as WWTP effluent in Fig. 4. The T2000 values were derived from these curves and using the respective calibration curves, the number of viable bacteria in each sample was calculated (Table 1).

**Table 1:** Number of bacteria (log CFU mL^−1^) and antibacterial activity (*A*) of QAS- and AgNPs-functionalized membranes on supporting layers (MFRF, VS, PP) and their respective non-functionalized control membranes

	*E. coli*				*E. faecalis*				WWTP effluent	
	Resazurin assay		Plate count assay		Resazurin assay		Plate count assay		Resazurin assay	
	log CFU mL^−1^ ± SD	*A*	log CFU mL^−1^	*A*	log CFU mL^−1^ ± SD	*A*	log CFU mL^−1^	*A*	log CFU_EEQ_ mL^−1^ ± SD	*A*
QAS-MFRF	8.06 ± 0.55	0.01	8.02	0.76	< LOQ	< 1	5.3	0.54	4.60 ± 1.13	0.31
PU-MFRF	8.07 ± 0.36		8.67		< LOQ		5.22		4.91 ± 0.71	
QAS-VS	9.13 ± 0.80	− 0.35	8.52	− 0.08	7.11 ± 0.63	0.25	7.09	0.36	6.49 ± 0.25	0.40
PU-VS	8.78 ± 0.64		8.44		7.36 ± 0.35		7.45		6.79 ± 0.22	
QAS-PP	9.68 ± 0.41	0.02	8.48	0.03	7.94 ± 0.06	0.02	7.71	0.49	7.63 ± 0.46	0.11
PU-PP	9.70 ± 0.27		8.51		7.96 ± 0.11		8.19		7.74 ± 0.44	
AgNPs-PP	< LOQ	> 6.70	3.27	5.24	< LOQ	> 4.96	3.45	4.63	< LOQ	> 4.74
PU-PP	9.70 ± 0.27		8.51		7.96 ± 0.11		8.19		7.74 ± 0.44	
Inoculum w/o membr.	9.95 ± 0.02	–	–	–	7.98 ± 0.25	–	–	–	7.72 ± 0.32	–

*A* was determined either by resazurin microplate assay or the plate count assay. *A* was calculated from Eq. 1 for resazurin assay and Eq. 2 for plate count assay and expressed as log CFU reduction compared to respective non-functionalized control membrane. A number of bacteria in the WWTP effluent sample was calculated from the *E. coli* calibration curve, therefore membrane *A* against WWTP effluent microbial community was expressed as a reduction of log CFU_EEQ_ mL^−1^

The membrane functionalized with AgNPs exhibited significant antibacterial activity with *A* > 5 (i.e. log CFU reduction compared to control non-functionalized membrane). In contrast, none of the QAS-functionalized membranes exhibited such a significant antibacterial activity compared to the corresponding non-functionalized control membranes, and *A* values were always below 1.

Nevertheless, the viable number of bacteria incubated with membranes was significantly lower than the number of pure bacteria without exposure to membranes regardless of functionalization or supporting layer (Fig. 4). It means the significant influence of supporting layer to the bacterial viability, which is especially pronounced in the MFRF supporting layer. For example, when compared a number of bacteria after the incubation with and without membranes, e.g. non-functionalized PU-MFRF delivers *A *= (9.95 − 8.07) = 1.88 for *E. coli* and *A * = (7.98 − LOQ being 3) = > 4.98 for *E. faecalis* (Table 1).

### Antimicrobial activity of membranes—plate count assay

The number of bacteria obtained from all membrane samples (both functionalized and non-functionalized membrane) at the beginning of incubation (T0) was 4.19 ± 0.13 log CFU per sample and 4.05 ± 0.35 log CFU per sample (i.e. 100 µL of original inocula), for *E. coli* and *E. faecalis,* respectively. These amounts yielded from membrane samples correspond to a bacterial inoculum of 1–3 × 10^5^ CFU mL^−1^ (1–3 × 10^4^ CFU 100 µL^−1^, corresponding log 4.00–4.47).

The membrane functionalized with AgNPs exhibited a significant antibacterial activity with *A* > 4. In contrast, QAS-functionalized membranes did not exhibit such significant antibacterial activity compared to the corresponding non-functionalized control membranes with *A* < 1. This is in a good agreement with the results of the resazurin assay (Table 1).

## Discussion

Several strategies for the assessment of antimicrobial properties of textiles and similar materials have been recently used, where the oldest one represented by the plate counting assay, is still the most common approach that is even standardized in ISO 20743 guideline. To develop faster and high-throughput alternative, we have optimized a resazurin microplate assay and used it successfully for the evaluation of the antibacterial activity of electrospun nanofiber membranes. Although we tested only PU membranes, the assay design can be used for assessment of any other filtration membranes or porous materials regardless of the type of material.

In the first step, the kinetics of resazurin metabolization in TSB suspensions with various initial bacterial concentrations was investigated and the metabolization curves were plotted (Fig. 2). Our results show that resazurin metabolization rate directly depends on the initial concentration of bacteria and their growth characteristics. However, when the amount of resorufin reached its maximum and entered a stationary phase, fluorescence became stable for certain time-period, while in the final part of each curve RFU values decreased. The decline was probably attributed to a secondary reduction of resorufin to colorless and non-fluorescent degradation product hydro-resorufin (Peeters et al. 2008). It was also observed that *E. faecalis* metabolized resazurin faster than *E. coli*, which is in a good agreement with Mariscal et al. (2009) study who also pointed out to interspecific differences.

To describe the relationship between bacterial concentration and a rate of resazurin metabolization, time periods to reach a level of 285 RFU (i.e. T285 values) by different bacterial concentrations was recorded. T285 was used in experiments in 96-well plates that were used for calibration curve measurements. This high-throughput setup enabled measurement of a whole range of bacterial dilutions at once, each concentration in hexaplicates for better accuracy. Another advantage was also lower consumption of resazurin and other solutions. However, the final membrane assessment was not feasible in 96-well plates because of the impossibility to prepare such small cuts of delicate nanofiber membranes. Thus, 12-well plates were used, and the amount of bacterial inoculum and resazurin solution were used in ten times higher volumes. Parallel experiments showed that such change of well size and the amount of bacterial inoculum and resazurin solution resulted in seven times higher RFU values. Thus, for the membrane assessments, the T2000 values were used for the calculation of the bacterial concentration from the respective calibration curve. The 285 or 2000 RFU level corresponds to the beginning of the exponential growth of resorufin concentration, which enables the most sensitive detection of possible antimicrobial effect in experiments with membranes. It should be noted that RFU values depend on the particular settings (especially the gain value), so the RFU values corresponding to the beginning to the exponential growth can be different at different fluorometers.

For both microorganisms, the LOQs were as low as 10^3^ CFU mL^−1^, which is equal to 10 CFU per sample because 0.01 mL of suspension per well was used in 96-well plates. This is comparable to limits of resazurin assay for planktonic *Salmonella typhimurium* and *Listeria monocytogenes* reported by Shiloh et al. (1997), while other authors stated that the concentration as high as 5 × 10^7^ CFU mL^−1^ or even higher was necessary to detect a significant fluorescence signal of planktonic *Staphylococci* (Sandberg et al. 2009). Resazurin assay was also previously found suitable to quantify microbial (either bacterial or yeast) biofilms. While applicable range determined by Peeters et al. (2008) was 10^6^ to 10^8^ CFU per well, Van den Driessche et al. (2014) succeeded in extending this range to 10^3^ to 10^8^ CFU per well.

Using the resazurin assay, a significant antimicrobial activity (*A*) of AgNPs-functionalized membrane (*A* was equal to 5 or even higher) was determined. Such a high antimicrobial activity is, however, not surprising, as AgNPs can target a broad spectrum of organisms (Marambio-Jones and Hoek 2010) and an antimicrobial potential of various kinds of water filtration membranes modified with AgNPs has been declared several times (Huang et al. 2016; Mecha and Pillay 2014; Parekh et al. 2018). On the other hand, *A* values for all QAS-functionalized membranes were lower than 1, which shows no significant antimicrobial activity of QAS itself. To confirm that the QAS is ineffective, pure QAS solution used for membrane functionalization was tested for antimicrobial efficacy by determination of minimum inhibitory concentration (MIC). Resulting MIC was 6% and 20% v/v for *E. coli* and *E. faecalis* respectively (unpublished results). As the amount of QAS used for membrane functionalization was only 5% v/v, it was insufficient for inducing the antibacterial effect. Although some authors previously prepared highly antimicrobial QAS-functionalized membranes (Kim et al. 2007; Meng et al. 2015), it was found that the antibacterial potential of the QAS depends on their chemical structure and the most potent QAS usually involve dialkyldimethyl substituents with a long length of alkyl chain (C_12_–C_18_) or substituents with the aromatic ring (Block 2000). The tested membranes were functionalized with bis{3,3′-[(6-hydroxyhexanoyl)amino]}-*N*-ethyl-*N*-methyldipropan-1-ammonium bromide, which does not possess any of these features.

Resazurin assay was also used for the assessment of antimicrobial activities of membranes against natural microbial communities of WWTP effluent. Since the microbial composition of WWTP-effluents can significantly vary in time and place, it was decided not to construct an own calibration curve for WWTP effluent, but to use the *E. coli* calibration curve, and express the number of bacteria in *E. coli* equivalents − CFU_EEQ_ mL^−1^. The results were very similar with the ones on pure bacterial species with a high *A* value for AgNP-functionalized membrane, but non-significant *A* values of QAS-modified membranes.

To validate the results of resazurin microplate assay, the assessment of antimicrobial activities using plate count assay was done as well. This assay was applied according to the modified absorption protocol under the ISO 20743 guideline intended for the determination of the antimicrobial activity of textiles. Modification of the protocol was in the selection of bacterial species *E. coli* and *E. faecalis* relevant for the aquatic environment instead of recommended *Staphylococcus aureus* and *Klebsiella pneumoniae*. Consequently, the incubation times were adjusted to the growth patterns of the selected species. Using the ISO guideline, we have obtained comparable results with the resazurin assay with a significant antibacterial effect for AgNPs-functionalized membrane and no significant effect for QAS-functionalized membranes (Table 1).

A counting of bacterial colonies on agar plates represents probably the most common approach to quantify bacteria in various samples and assess possible antimicrobial effects (Allen et al. 2004; Van Nevel et al. 2017). The main advantage involves well-standardized protocols and usually higher sensitivity compared to other methods. As the relatively narrow range of bacterial colonies (25 to 250) has been considered countable, LOQ for plate count assays is 25 CFU mL^−1^ (Sutton 2011), which is about one order of magnitude lower than in the case of the resazurin assay. On the other hand, the resazurin assay enables an analysis of microbial biofilms without necessity of sonication or any other complicated sample pre-treatment. This aspect is undoubtedly important, especially in the case when the tested membranes are gentle and vulnerable to damage. The limitations of this method are a necessity to calibrate each bacterial strain first, however, in case of working with mixed species communities, it could be advantageous to express the number of bacteria in equivalents of other common species like *E. coli* in our case.

In conclusion, the resazurin microplate assay has been suggested and optimized for the evaluation of antimicrobial properties of electrospun nanofiber filtration membranes. Using different regression models, the calibration curves describing a relationship between concentrations of either *E. coli* or *E. faecalis* in bacterial suspension and a rate of resazurin metabolic reduction to resorufin were obtained. Low LOQs values, 1.0 × 10^3^ CFU mL^−1^, were achieved for both microbial species. The resazurin assay demonstrated the significant antimicrobial activity of the membrane functionalized with AgNPs against both laboratory bacterial cultures as well as a natural microbial community of WWTP effluent. Although our results suggested a weak antimicrobial activity of membrane functionalized with QAS that possessed MFRF supporting layer, the effect was probably caused by supporting layer itself rather than attributed to the functionalization of nanofiber polymer matrix with QAS. These results were in a good agreement with a conventional plate count assay. The resazurin assay seems to be a good alternative to plating technique, as it is faster, and its microplate design allows automatization which represents a crucial step for possible high-throughput analysis. The method enables an analysis of planktonic microorganisms, and also biofilms, without necessity of sonication or another pre-treatment step, which is very important, especially when the work with membranes of low mechanical resistance is needed.

## Supplementary information

**Additional file 1.** Additional Materials S1, S2, S3

## Data Availability

All the relevant data and materials are published in the article or in additional file.
