# Supplementary material for: Resazurin assay for assessment of antimicrobial properties of electrospun nanofiber filtration membranes
Source: AMB Express. 2019 Nov 13;9:183. doi: 10.1186/s13568-019-0909-z (PMC6854189; doi:10.1186/s13568-019-0909-z)
Supplement: Supplementary file 1 — Additional file 1. Additional Materials S1, S2, S3 [file 13568_2019_909_MOESM1_ESM.doc]

**AMB Express**

Resazurin assay for assessment of antimicrobial properties of electrospun nanofiber filtration membranes

Eva Travnickova^1^, Premysl Mikula^1,2^, Jakub Oprsal^3^, Marie Bohacova^3^, Lubomir Kubac^4^, Dusan Kimmer^5^, Jana Soukupova^6^, Michal Bittner^1*^

^1^ Research Centre for Toxic Compounds in the Environment, Masaryk University, Faculty of Science, Kamenice 5, 625 00 Brno, Czechia

^2^ Department of Veterinary Public Health and Forensic Medicine, University of Veterinary and Pharmaceutical Sciences Brno, Palackeho 1946/1, 612 42 Brno, Czechia

^3^ SYNPO a. s., Pardubice, S. K. Neumanna 1316, 532 07 Pardubice, Czechia

^4^ Centre for Organic Chemistry Ltd., Rybitvi 296, 533 54 Rybitvi, Czechia

^5^ Centre of Polymer Systems, University Institute, Tomas Bata University, trida Tomase Bati 5678, 760 01 Zlin, Czechia

^6^ Regional Center of Advanced Technologies and Materials, Department of Physical Chemistry, Palacky University, Šlechtitelů 27, 783 71 Olomouc, Czechia

*Corresponding author: E-mail: bittner@recetox.muni.cz, Phone: +420 549493807

Additional file 1: Material S1

Growth of bacteria inoculated to the TSB media

The ISO 20743 guideline stated 180 min incubation time for preparing the cultures. According to the guideline, the point is to incubate the organisms as long as they get into the exponential growth phase. Different organisms than recommended in the guideline were used, so the exact time of their exponential growth phase was measured.

Experimental organisms *Escherichia coli* and *Enterococcus faecalis* were grown to the second subculture (see chapter 2.2 of the article). 400 μL of the second subculture were transferred into 20 mL of TSB and incubated at 37 °C on an orbital shaker at 110 rpm (rounds per minute). Then, the OD_600_ was measured every 30 minutes. For *E. coli*, two independent measurements were performed. For *E. faecalis*, one measurement was performed.


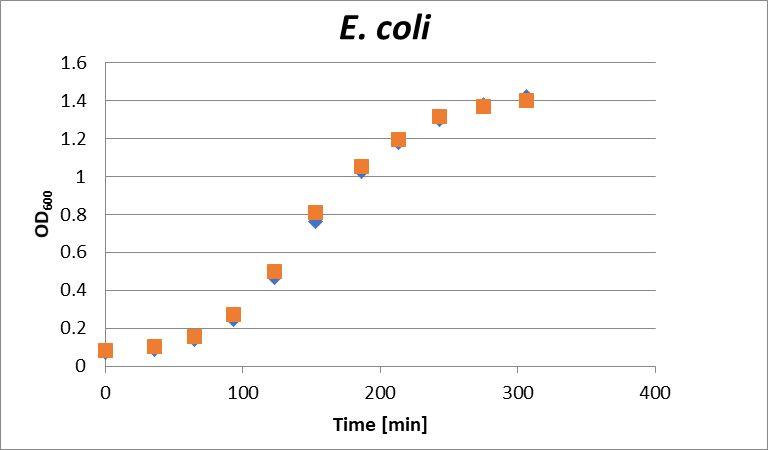


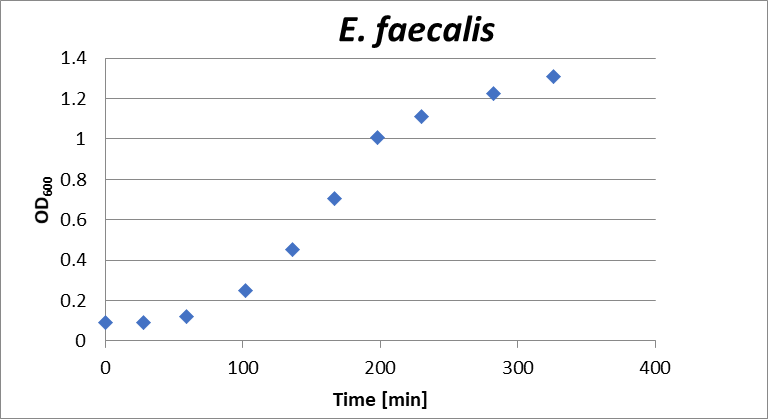


Additional file 1: Material S2

Description of the source of WWTP effluent sample

The wastewater treatment plant (WWTP) effluent flows directly to the Ochozsky stream, which is a periodically flowing stream. During the dry seasons of the year, usually in Summer and Winter, the WWTP effluent is only the source of water in the stream.

The WWTP is treating the wastewater from the Březina municipality where lives about 1050 inhabitants (January 2019). There is no major industry in the municipality. There are two separated sewerage systems for wastewater and for rainwater in the municipality where wastewater flows to the WWTP and the rainwater directly to the Ochozsky stream.

The wastewater entering the WWTP is treated mechanically at first by separation of solid particles, and consequently treated biologically with denitrification and then nitrification processes. The organic compounds are bacterially degraded together with nitrogen removal. The nitrification step can be operationally extended with the precipitation of phosphorus with ferric sulfate.

GPS coordinates of the sampling place: 49.2765303N, 16.7450597E

Picture of the sampling place taken from:

[https://mapy.cz/zakladni?vlastni-body&x=16.7416373&y=49.2758512&z=15&base=ophoto&ut=Nov%C3%BD%20bod&uc=9mgSEx8RB8&ud=49%C2%B016%2735.509%22N%2C%2016%C2%B044%2742.215%22E](https://mapy.cz/zakladni?vlastni-body&x=16.7416373&y=49.2758512&z=15&base=ophoto&ut=Nový bod&uc=9mgSEx8RB8&ud=49°16'35.509"N%2C 16°44'42.215"E)

**
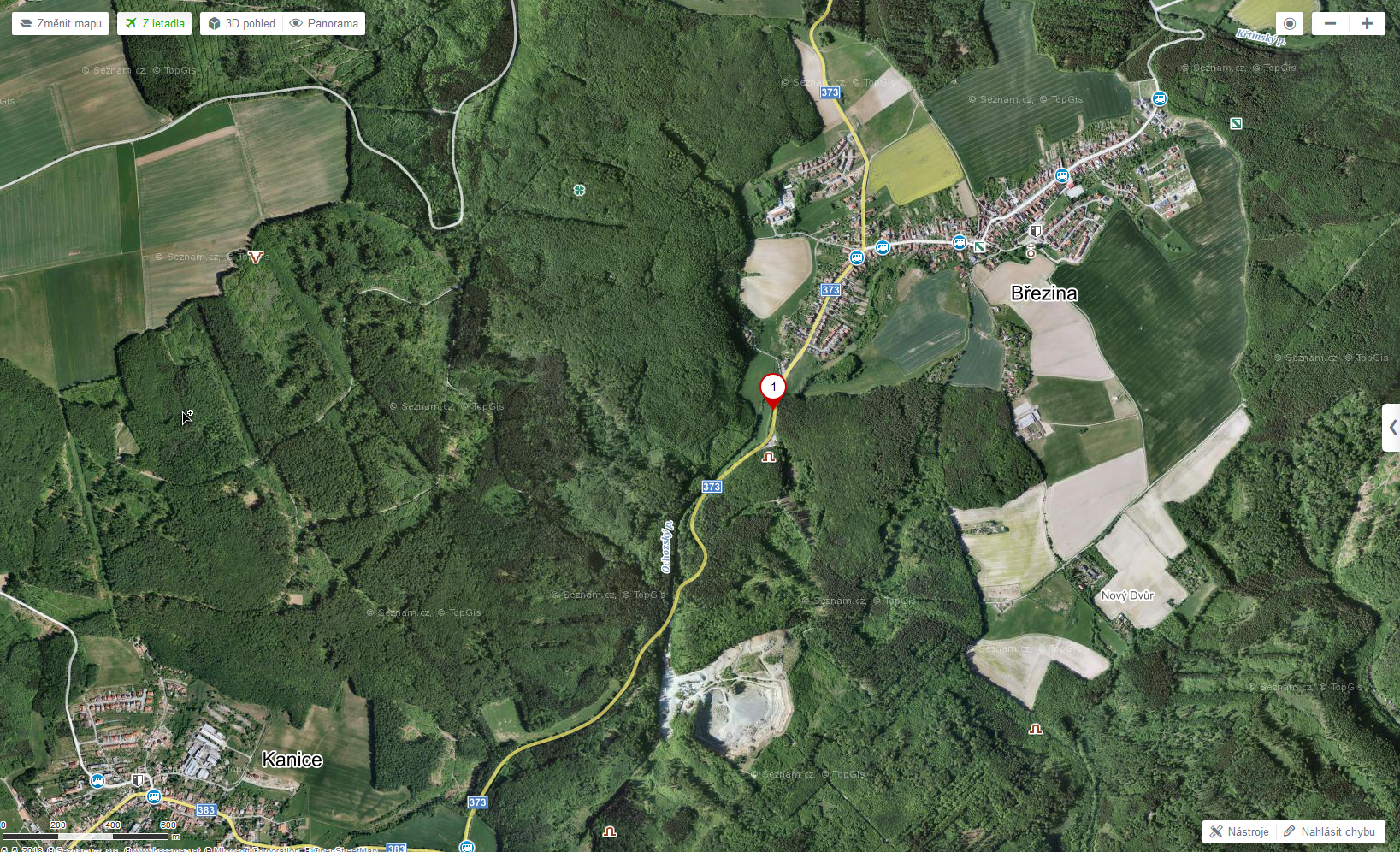
**

Additional file 1: Material S3

Explanation of conversion of results obtained by using 96-well plate to the results obtained by using 12-well plate

For the experiments with the actual membranes, we have changed the experimental microplates from 96-well plate (used for calibration curves establishment) to 12-well plate. The change of well-plate size and liquids volumes was necessary due do practical reasons (suitable size of membrane cuts and stability during manipulation). Because of these issues, the relation between fluorescence signals from 96-well plate (used for setup of calibration curves) and 12-well plate was measured; it is referred to as “well conversion factor” in subsequent calculations. In the case of the 12-well plate, 100 µL of bacterial suspension and 2 mL of resazurin were used, compared to the 96-well plate which included 10 µL and 200 µL; all other parameters remained unchanged. The fluorescence of generated resorufin was recorded in the same way in both 96- and 12-well plates in the parallel measurements. The fluorescence values from the 12-well plate were divided by fluorescence values from the 96-well plate. The resulting conversion factor between the fluorescence signals from the 96-well plate and 12-well plate was 7. The conversion factor is independent of bacterial species. All the subsequent results obtained in 96-well plates presented in this paper have already been recalculated by multiplication with conversion factor 7 to fit the fluorescence values obtained from the 12-well plates.
